# Osteolysis: A Literature Review of Basic Science and Potential Computer-Based Image Processing Detection Methods

**DOI:** 10.1155/2021/4196241

**Published:** 2021-10-04

**Authors:** Soroush Baseri Saadi, Ramin Ranjbarzadeh, Ozeir kazemi, Amir Amirabadi, Saeid Jafarzadeh Ghoushchi, Oveis Kazemi, Sonya Azadikhah, Malika Bendechache

**Affiliations:** ^1^Department of Electrical Engineering, Islamic Azad University, South Tehran Branch, Tehran, Iran; ^2^Department of Telecommunications Engineering, Faculty of Engineering, University of Guilan, Rasht, Iran; ^3^PPD - Global Pharmaceutical Contract Research Organization, Central Lab, Zaventem, Belgium; ^4^Faculty of Industrial Engineering, Urmia University of Technology, Urmia, Iran; ^5^Mehr Parand Clinic, Parand, Tehran, Iran; ^6^R.E.D. Laboratories N.V./S.A., Z.1 Researchpark, Zellik, Belgium; ^7^School of Computing, Faculty of Engineering and Computing, Dublin City University, Dublin, Ireland

## Abstract

Osteolysis is one of the most prominent reasons of revision surgeries in total joint arthroplasty. This biological phenomenon is induced by wear particles and corrosion products that stimulate inflammatory biological response of surrounding tissues. The eventual responses of osteolysis are the activation of macrophages leading to bone resorption and prosthesis failure. Various factors are involved in the initiation of osteolysis from biological issues, design, material specifications, and model of the prosthesis to the health condition of the patient. Nevertheless, the factors leading to osteolysis are sometimes preventable. Changes in implant design and polyethylene manufacturing are striving to improve overall wear. Osteolysis is clinically asymptomatic and can be diagnosed and analyzed during follow-up sessions through various imaging modalities and methods, such as serial radiographic, CT scan, MRI, and image processing-based methods, especially with the use of artificial neural network algorithms. Deep learning algorithms with a variety of neural network structures such as CNN, U-Net, and Seg-UNet have proved to be efficient algorithms for medical image processing specifically in the field of orthopedics for the detection and segmentation of tumors. These deep learning algorithms can effectively detect and analyze osteolytic lesions well in advance during follow-up sessions in order to administer proper treatments before reaching a critical point. Osteolysis can be treated surgically or nonsurgically with medications. However, revision surgeries are the only solution for the progressive osteolysis. In this literature review, the underlying causes, mechanisms, and treatments of osteolysis are discussed with the main focus on the possible computer-based methods and algorithms that can be effectively employed for the detection of osteolysis.

## 1. Background

Basically, osteolysis is a biological process initiated by induced particles at the interface of bone metal or bone cement of prosthetic implants which is radiographically manifested as linear endosteal radiolucencies or scalloped focal. This phenomenon in long-term results in bone loss, periprostatic fractures, and finally loosening of implants. From early observations, it was generally believed that, in cemented implants, osteolysis is due to acrylic cement and the term “cement disease” was introduced because of this belief. Nevertheless, osteolysis is now defined as “particle disease” with the demonstration of lytic lesions in implants without cement in the interface [1–3].

Osteolysis is one of the main causes for late reoperation in patients with total joint arthroplasty (TJA). This operation is the most effective therapeutic solution for patients enduring end-stage degenerative arthritis. It is believed that the demands for TJAs will gradually increase worldwide in subsequent years where total hip arthroplasty (THA) and total knee arthroplasty (TKA) are the most frequents types. Although the lifetime of THA has improved, such that approximately 90% of the implants function appropriately at 15 years [4], osteolysis is the main cause of at least 50% of all THA revision surgeries based on the majority of national registries. Despite the fact that the effectiveness of surgery may depend on various variables including the level of physical activity of patients, type of implant material, time of THA in service, model of the implant and placement [5], more than 100,000 patients for each million THA procedures might experience a revision surgery within a 15-year period of service. Osteolysis has been noted in both cemented and cementless implants, with 0% to 16% for cemented and 6% to 30% for cementless TJA.

**Figure 1:**
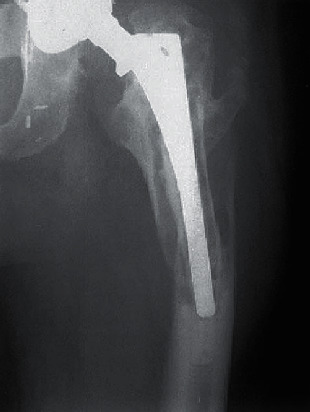
Periprosthetic femoral osteolysis caused by wear of an extended-chain crystallite polyethylene [10].

It is indicated by clinical experience that there is a strong correlation between the likelihood of osteolysis and the magnitude of polyethylene [6]. The majority of the wear particles are ultrahigh molecular weight polyethylene (UH-MWPE) which is mainly implicated in the development of lesions [7–9]. The appearance of osteolysis at shorter times of follow-up will become more apparent as the higher wear rates cause higher rates of osteolysis [10] (Figure 1). Considering a uniform distribution of wear particles through the periprosthetic tissue, the wear volume can be directly related to the number of particles per unit volume of tissue [11]. Nevertheless, it is improbable that the distribution of wear particles is uniform as the permeability of tissues varies, and there are also a small number of pathways for particle access. Local osteolysis can be produced as a result of local accumulation of wear debris even if the general density of particles is low. This makes it difficult to determine the relationship of appearance of osteolysis to wear.

Osteolysis is a progressive medical condition. Thus, regular follow-up sessions are advised. During these sessions, orthopedists check the progress of the condition with the use of different imaging modalities depending on the severity of the condition. Nevertheless, regardless of imaging techniques, detection and analysis of osteolysis are difficult tasks to perform as osteolytic lesions are not easy to be distinguished especially at the first years after implantation. This is the main reason that researchers and biomedical engineers in the field of medical image processing are making a great effort to devise new methods and algorithms to overcome these barriers.

In recent years, artificial intelligence (AI) has become highly popular and is developing into every aspect of the modern life by its advances in large-data retrieval and explicit evaluation of features that are ideal for medical image processing [12–15]. In comparison to traditional image processing methods, deep learning is more efficient and reliable, as it can automatically extract features of the images instead of hand-crafted features [16, 17]. Thanks to the deep neural networks (DNNs), the methods of computation can allow an algorithm to self-programme through learning from a large set of examples that show the desired behavior, eliminating the need for specifying the rules explicitly [18,19]. Deep learning (DL) techniques have developed fast and have been shown to exceed the performance of human beings. Deep convolutional neural networks (DCNNs) demonstrate a great benefit in image processing. CNN is a subclass of deep, feedforward neural networks, in which image data moves in a forward direction from the input to the output nodes. The advantageous feature of CNNs is that they can learn directly useful image features and other structured data, whereas the task of feature extraction has been carried out by machine learning models or by hand before CNNs. These types of neural network architecture proved to be powerful deep learning models in the field of image analysis, judging from the existence of specific features in their structure [20–22]. A typical CNN is composed of one or several filters that are called filters, kernels, or convolutional layers, accompanied by several layers (an aggregation and pooling layer) that are employed for classification purposes. As the characteristics of CNN are similar to those of the standard artificial neural network (ANN), backpropagation and gradient descent are used for training tasks, while it is comprised of additional pooling layers along with kernels. The final results are derived from the vector that is sited at the end of the network architecture. CNNs can have various structures and methods. In medical image analysis, the most popular structures are U-Net [23], SegNet [24], and V-Net [25] as well as the conventional CNN structures. Additionally, the most common CNNs methods are the following: ZFNet (2013) [26], VGGNet-16 (2014) [27], AlexNet (2012) [28], GoogLeNet [29], DenseNet (2017) [30], and ResNet (2015) [31]. There are several studies that prove the effectiveness of CNN algorithms in medical image processing, such as breast cancer detection [30], diagnosis of breast ultrasonography images [32], liver cancer detection and segmentation [33], differentiation of liver masses [34], lung infection segmentation [35,36], and classification of interstitial lung disease [37]. However, one key drawback of most deep learning approaches is that they need a lot of training data to learn features properly.

The intent of this study was not to investigate comprehensively the biological and medical aspect of osteolysis. An attempt was made, however, to give a guideline for the main factors and issues involved in this medical phenomenon and to introduce computer-based methods and algorithms especially in the field of artificial neural networks used successfully for similar purposes.

## 2. Discussion

### 2.1. The Biological Mechanisms of Osteolysis

**Figure 2:**
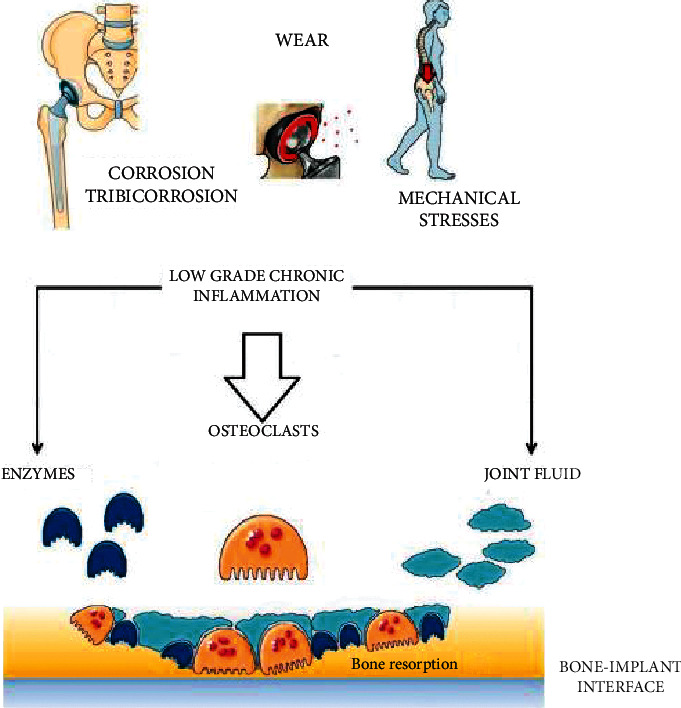
Amplification and translation of biological signals start with the interaction of prosthetic bearing wears with the human innate immune system cells resulting in bone resorption in bone multicellular units at the interface of the bone implant [1].

Osteolysis is an active biological response to particulate wear debris [38], primary bone tumors [39], and metastatic diseases [40]. In fact, this phenomenon is a cell-mediated biological process leading to bone loss as a direct reaction of stimulation of macrophages by biologically active particles. It is noted that the mechanical wear of the articulating surface that releases wear debris begins this process. This key debris is phagocytosed, which activates osteoclasts and macrophages and results in resorption of the bones [3] (Figure 2).

The cells which are mainly involved in creating a response to the particles are fibroblasts, lymphocytes, macrophages, osteoclasts, osteoblasts, and foreign body giant cells. The main cellular mediator of osteolysis is macrophages that take part individually in the resorption of bones by transforming into osteoclasts leading to much faster resorption. These cells secrete a variety of cytokines and are present in the pseudomembrane. Among them, the most important cells are interleukin 6, interleukin 1*α*, interleukin 1*β*, TNF-*α*, prostaglandin E2, RANKL, gelatinase, and collagenases [1, 41–43].

Generally, we can distinguish between nonwear-related and wear-related byproducts. At each stage, wear particles are released from the surface of the softer material by abrasion and adhesion of bearing surface [44]. During the time of service, it is recognized that all lower-limb arthroplasties generate wear particles [45]. Based on the biotribological experiments, materials which are hard similar to ceramics that create nanosized wear particles can cause inflammation [46]. Bone cement debris and metallic debris were also noted to have relation to hypersensitivity and inflammation [47–49]. Nevertheless, there is limited evidence on the influence of the prosthetic particle dimension, surface charge, shape, and osteoclast capacity. The experimental evidence also demonstrates that erosive bone resorption can cause prosthetic joint infection if not diagnosed and treated early [50–52].

It has also been recognized that local hydrodynamics factors can contribute to osteolysis. Local fluid pressure gradients around implants are considered to create a mechanism for the particle to transport and force fluid and to surround the bone. Very high intra-articular pressures, caused by fluid pressure, changes during motion can mount osteocyte death and subsequently induce osteoclast bone resorption [53].

Finally, it is also noted that the type of metal debris has a profound consequence for the extent of osteolysis. Titanium has a greater destructive stimulatory impact than cobalt-chromium (CoCr). Early death of the macrophages caused by CoCr particles reduces the inflammatory mediators, leading to osteolysis. On the other hand, titanium debris is less toxic to the macrophage, thus triggering upper levels of the inflammatory mediation [54]. In addition, experiments have demonstrated that the mean particle dimension of less than 1.7 ± 0.7 um of wear particulate debris will increase the rate of osteolysis after THA [55].

### 2.2. Types of Osteolysis

Generally, there is no agreement on the classification of osteolysis as different methods of measurements are used in experiments and analysis. Although many studies have been conducted on this phenomenon, normally the incidence is reported. In fact, for the classification of osteolysis, lesion volume is measured. This is carried out by measuring the area of the osteolytic regions on lateral and anteroposterior radiographs. In some cases, only one view is used. On plain radiographs, normally femoral lesions can be easily viewed, whereas this is not the case for acetabulum lesions. Revision surgeries have shown that the lesions behind the acetabulum are larger than the dimension expected from preoperative radiographs [56, 57].

Not all osteolytic lesions (softened section of a patient's bone) lead to failure although they are considered as an unfavourable medical issue and are a predictive factor for later adverse consequences. There are some osteolytic lesions that are stable and do not expand, whereas there are others which have a balloon-like features and can expand and propagate [58, 59]. Osteolytic lesions can diffuse or be localized. The diffuse lesion can progress along the interface or be stable. Depending on the location, the prognosis for a lesion can vary. Although the lesions behind the acetabular component may not progress, they are naturally expansive at this location [60].

**Figure 3:**
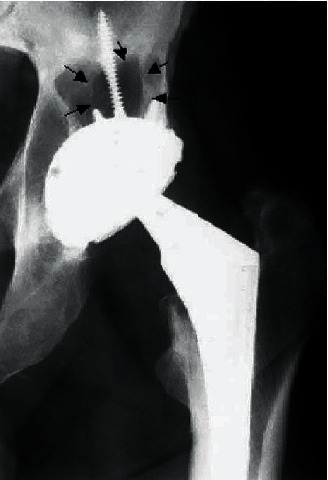
Osteolytic lesion superior to the well-fixed modular acetabular component [62].

**Figure 4:**
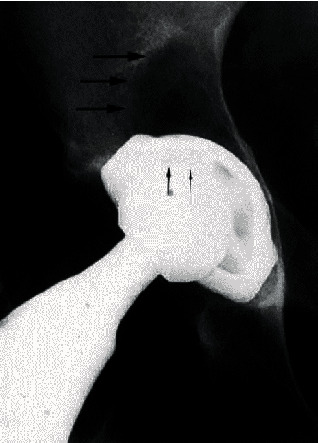
Osteolytic lesion superior to the loose press-fit modular acetabular component [62].

Furthermore, it seems also that cemented and cementless implants give rise to different biological processes [61]. In cemented acetabular components, there is a tendency to show a pattern of osteolysis leading to losing the implant, whereas cementless components tend more to exhibit expansile, localized lesions with the cup of the implant remaining stable (Figure 3) [62]. Additionally, although loosening of the component of the implant may precede the progress of an expansile lesion, it is less often (Figure 4).

### 2.3. Diagnosis

The most popular method of detection of the extent of osteolytic lesions is computed tomography whereas the most sensitive method is magnetic resonance imaging (MRI). The studies have demonstrated that the sensitivity of CT and plain radiography in the detection of osteolysis is 74.7% and 51.7%, respectively, while the corresponding figure for MRI is 95.4% [63]. Furthermore, thanks to MRI images, it is not only possible to detect intracapsular synovial disease well before discernible loss of bone, but also to detect the granulomatous tissues caused by the wear debris, which are actually the merits of this tomographic modality [64].

Conversely, it is a difficult task to identify osteolytic lesions on plain radiographic images because of two reasons. First, the visualization of the cancellous bone adjacent to the prosthesis can be blocked by the tibial and femoral components. Second, before detecting any changes on the quantity of the skeletal calcium on the radiographic images, 50% of them are normally lost [65].

### 2.4. Methods of Diagnosis

Currently, various methods are employed for the detection and analysis of the osteolytic lesions. However, the application of these methods is highly dependent on the knowledge of the medical profession, available facilities, and severity of the condition. Diagnosis of the osteolysis can be implemented manually using radiographic, CT, and MRI images or can be carried out with semiautomatic or automatic computer-based techniques, algorithms, toolkits, and software. Recently, thanks to the advances in the artificial neural networks, deep learning algorithms are widely used by orthopedists and medical profession with the assistance of biomedical research for the detection of a variety of bone tumors and fractures, analysis of prosthetic movements, etc. Although fewer computer-based studies have been carried out specifically on the topic of osteolysis as there are very limited public datasets for this topic, because of strict resemblance between anatomical regions of interest, tissue properties, biological behaviors, detection, and analytic modalities, the majority of these methods are applicable for the analysis and detection of osteolysis.

Following the most recent and state-of-the-art potential computer-based methods from semiautomatic to automatic methods, software and algorithms that can be employed to assist medical profession in the analysis of osteolytic lesions are briefly discussed.

#### 2.4.1. Deep Learning Methods

**Figure 5:**
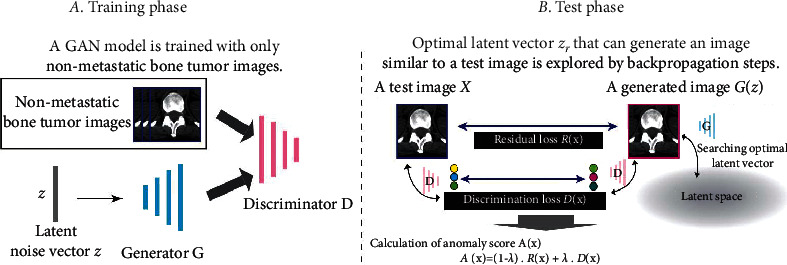
An overview of the generative adversarial network with anomaly detection of the bone tumor [67]. (a) Training phase. (b) Test phase.

*(1) Method 1*: *AnoGAN*. It is important to detect osteolytic lesions in advance during the follow-up sessions before they reach to a level leading to surgical treatments. Since osteolytic lesions are very small and have abnormal shape from unpredictable adjacent areas in the bone, the image collection with osteolytic lesions is challenging compared to major cancers. In this case, it can be determined that abnormality recognition is a proper idea. In the anomaly detection, the network is trained with a large number of images without lesions and then judges whether images show lesions or not based on a determined abnormality score [66]. As the number of images with lesions is limited in comparison to the number of images without lesions, the anomaly technique could be an efficient solution. Although labeled images are used for training the network, it is a heavy burden for clinicians to annotate the images. In order to reduce the labor for medical professionals, unsupervised learning methods are normally considered. This is due to the fact that all CAD systems can be more helpful in detecting a target more accurately in the image. Watanabe et al. [67] carried out a study using a similar modality and called it AnoGAN, which can be useful in detecting osteolytic lesions. They designed a classification method that functions based on adversarial learning but used bone tumor datasets to detect the metastatic bone tumors. Tumors lesions are distinguished based on computed abnormality scores. These scores are defined by comparing a generated image with a test image in the image level and the future level. The suggested strategy takes only nonmetastatic bone tumor images and learns the normal image distributions based on a generative adversarial network (GAN). Although using unsupervised learning method is quite challenging in medical image processing, it is a significant task for clinical applications.  Figure 5 demonstrates the anomaly detection framework proposed by Watanabe et al. [67].

**Figure 6:**
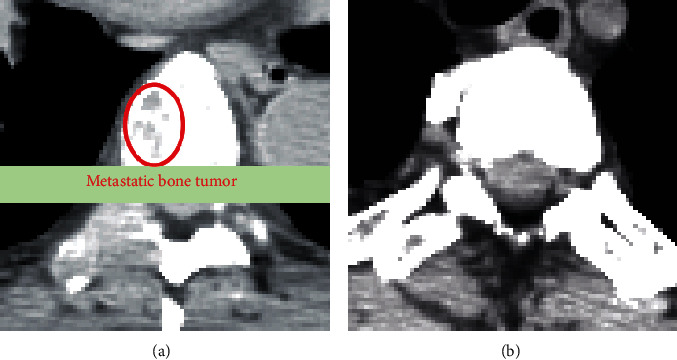
Image samples of true positive (a) with metastatic bone tumor and true negative without tumor (b) detected by the AnoGAN method [67].

As can be seen from Figure 5, the proposed method is composed of two steps: training step and test step. During the training phase, the generative *G* and discriminator *D* networks compete to gain ability. After adversarial training, the generator network is able to produce nonmetastatic bone image from a latent noise vector *z*. This trained generator and the noise vector *z* are then used for the evaluation of the test samples in the next phase. The test phase employs the latent space to evaluate test samples. When a test image is imported, the method finds the finest *z* corresponding to the image *G*(*z*) generated by the generator network. The *z* value should be visually similar to *X* in iterative backpropagation stages. In the latent space, the best noise vector *z* can be found. The anomaly score is obtained with the following formula:

(1) $$\begin{array}{c} A\left(X\right)=\left(1-\lambda \right).R\left(X\right)+\lambda .D\left(X\right), \end{array}$$

where *D*(*X*) represents the discrimination loss that calculates the distinction of extracted features using the trained discriminator *D* in the features level. *R*(*X*) denotes the residual loss that computes the visual dissimilarity between *G*(*z*) and *X* in the image level. *λ* represents a weighted coefficient. The larger the value for the anomaly score, the greater the probability of anomaly detection.  Figure 6 shows the experimental results using this method.

**Figure 7:**
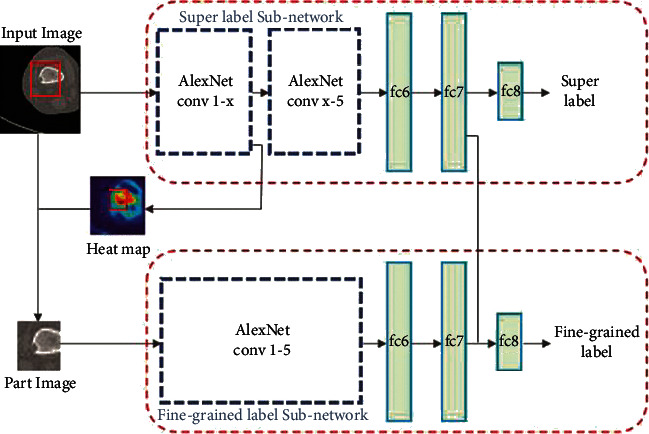
The superlabel-guided convolutional neural network (SG-CNN) structure. A raw CT image with two labels without any annotations is fed into the model. The input image is cropped under the guide of the heatmap generated by the first convolutional layer of the superlabel subnetwork and then inserted into the other network branch. The network output provides two labels [71].

*(2) Method 2*: *SG-CNN*. CNNs are very popular for classification tasks. There are various classic models of CNN such as VGGNet [27] and AlexNet [68] that can be selected for categorization tasks. Although these networks demonstrate good performance for classification of natural images, they are not very efficient for classification of medical images especially when the goal is to detect tumor lesions like osteolysis. The reason is that, in natural images, most objects are normally at the center of image and the variation between objects is apparent, whereas medical image categorization demands fine-grained visual classification that cannot be done with classic CNNs with high classification accuracy. Although several methods are proposed to overcome the fine-grained labeling problem such as a mask-CNN structure based on annotations of part of images by Wei et al. [69] and part-based R-CNN structure for fine-grained labeling by Zhang et al. [70], these methods are time consuming on making datasets. To solve this problem, a novel CNN is proposed by Li et al. [71]; it can produce ROI areas automatically by network independently without employing annotated images (Figure 7).

**Figure 8:**
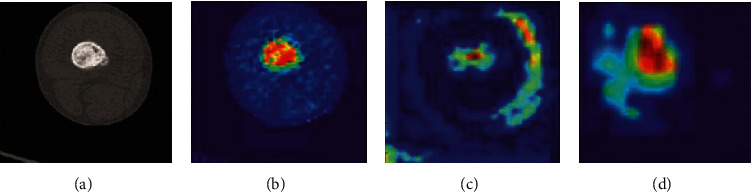
The presentation of raw data and heatmap. (a) A typical input image. (b) The heatmap generated by conv1. (c) The heatmap produced by conv2. (d) The heatmap generated as the output of conv3. It is determined by the images that the more we go deeper into the network, the more the abstract and semantic meanings contained in the heatmap [71].

This model is a superlabel-guided convolutional neural network (SG-CNN) that classifies CT images of bone tumor. SG-CNN is composed of two subnetworks that are responsible for learning the whole image and focus on bone tumor lesions to learn more detailed data. The inputs of these networks are CT images with two labels in hierarchical relationship, and the outputs are fine-grained labels and superlabels that both are used for training the SG-CNN to gain classification accuracy. AlexNet CNN model was used as the network architecture. During training phase, images are fed into the superlabel subnetwork. When all the feature maps of the guide convolution layer of the subnetwork are added together, a heatmap is generated (Figure 8). The heatmap generated during this phase, superimposed on the points corresponding to the image parts, is used as the input of the fine-grained label subnetwork. As the image is cropped, most of the background parts of the image are removed. Thus, the model emphasizes the potential tumor areas. Finally, the output of the network predicts fine-grained label whose classification accuracy is considered by two structure branches simultaneously.

Employing this artificial neural network model could effectively be used for the classification of osteolytic lesions, as the same obstacles are involved in the detection and classification of bone tumors, and the appearance of osteolytic lesions in CT images is very similar to that of bone tumors. Considering the successful results obtained using a bone tumor dataset on the SG-CNN by Li et al. [71], this method could be highly reliable.

*(3) Method 3*: *U-Net*. Convolutional CNNs used for the classification are composed of convolutional layers followed by several fully connected layers that map the feature image produced by kernels into a fixed-size feature vector [72]. Nevertheless, one of the defects of CNN is that each time a convolutional operation is accomplished for the classification, feature map resolution is diminished by half. This can reduce the accuracy of the classification if the feature map achieved by the final classification is utilized. Therefore, if convolutional neural networks are used for the classification of osteolysis, CNN may cause transition during the convolution operation. Although the performance of the model is rarely affected, the position of osteolytic lesions may simply be affected in the recognition step. Considering the fact that osteolytic lesions have abnormal shape and low contrast, this defect can visually affect the images by metallic artifacts and image noise [12, 25, 73, 74].

**Figure 9:**
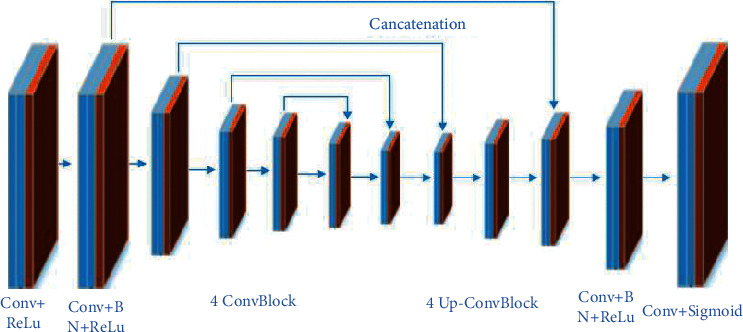
End-to-end network structure [75].

In comparison to the CNN, the fully convolutional layers in the fully convolutional networks (FCN), which accept input data (images) at any dimension, replace all the convolutional layers. In this network, a deconvolution layer is applied in order to perform upsampling for the feature map of the last convolutional layer to be at the same dimension of the input 2D data (image). Thus, while preserving the spatial information in the original input image, a prediction can be made for each pixel. Finally, the pixel-by-pixel classification can be performed on the upsampled feature map (Figure 9). Based on the residual network strategy, the FCN can solve the contradiction between the translation invariance in the target detection step and the translation invariance in the classification network. Thus, a semantic segmentation structure U-Net based on fully convolutional neural network can be an efficient method for the detection and classification of osteolytic legions.

**Figure 10:**
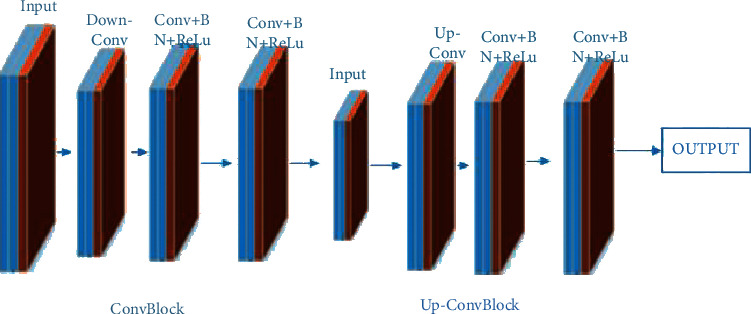
The U-Net network architecture. The contracting path is composed of two 3 × 3 convolution layers for repetitive processing, each accompanied with a modified linear unit (ReLU). The downsampling is composed of a single 2 × 2 maximum pooling operation [75].

In a study carried out by Jian et al. [75], a U-Net neural network consisting of a contraction path and an expanding path (Figure 10) was used for the diagnosis of osteoporosis, which is the most common bone disease [76, 77]. For the detection of osteoporosis of the femoral neck, if a conventional CNN is employed, it could cause translation during the convolution operation. This could easily affect the position of the osteoporosis boundary box in the detection step that directly leads to a reduction in the segmentation accuracy of the femoral neck.

**Figure 11:**
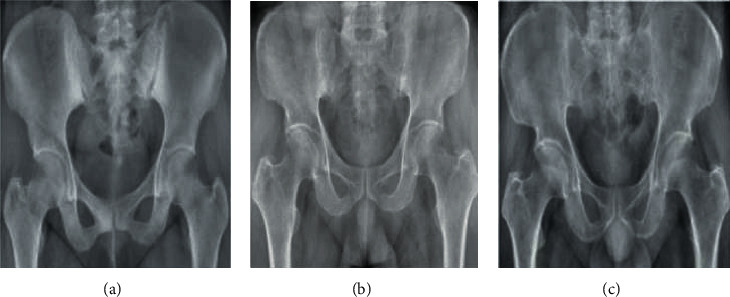
Some examples of pelvis acquired by X-Ray imaging: (a) normal bone mass, (b) lower bone mass, and (c) osteoporosis [75].

Osteoporosis is normally specified by a reduction in the bone density, thinning of cortical bone, and thinning of trabecular bone. The U-Net network proposed by Jian et al. [75] was implemented on an image dataset of patients having undergone pelvic X-ray imaging. Among the patients, 30 had normal bone mass, 28 had lower bone mass, and 31 had osteoporosis. Normal bone mass X-ray images demonstrate thick cortical bone and high bone density (Figure 11).

The recognition results and classification of the U-Net network proposed by Liu et al. are as follows: the total recognition rate of lower bone mass images from normal bone mass is 83.88%, the corresponding figure for the osteoporosis from normal bone mass is 86.74%, and the one for osteoporosis from lower bone mass is 79.55%. The experimental results of the network demonstrate that it can successfully solve the influence of image interference for the bone density analysis. The proposed U-Net network with the recognition rate of above 81% for the detection of osteoporosis could be a highly functional solution to solve a similar problem for the detection of osteolysis.

*(4) Method 4*: *Seg-UNet*. Do et al. [78] proposed a novel method for the detection of knee bone tumors from X-ray images using a multilevel Seg-UNet model with global- and patch-based techniques. This network is used as a computer-based assistive tool for the segmentation and classification of tumor regions into three labels: normal, benign, and malignant. Although this network is designed only for bone tumor detection around knee regions, as the anatomical region is at the same place where osteolysis will occur after TKA and there are similar visual characteristics in X-ray images, developing this network can effectively solve the problem of segmentation and classification of osteolytic lesion.

**Figure 12:**
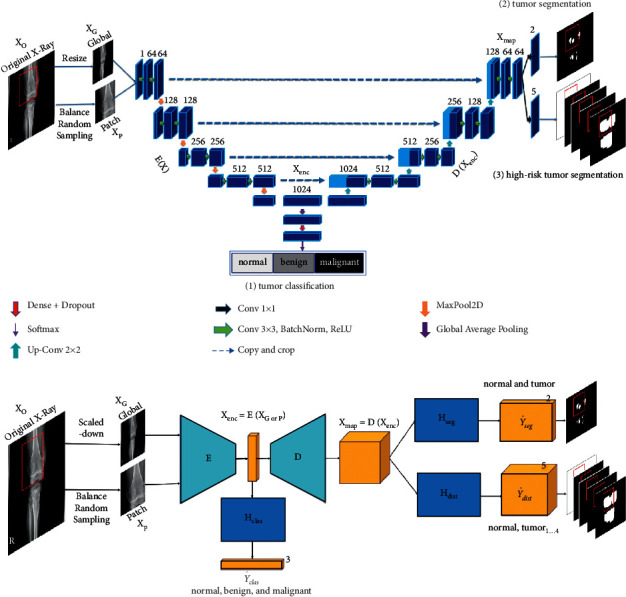
The multilevel Seg-UNet model with patch- and global-based approaches (top) and the block demonstration of the model (bottom). In the block diagram, E represents the encoding block; D depicts the decoding block; the three branch blocks H_seg_, H_dist_, and H_clas_ denote multitask learning [78].

The proposed Seg-UNet architecture is illustrated in Figure 12. This multilevel network uses a combined global- and patch-based approach in order to not only detect small tumor regions but also achieve a high improvement in malignant tumor detection.

This model has an encoder-decoder architecture to exploit the mutual advantage of segmentation and classification branches to learn the local texture features and global geometric context at every pixel. The encoding block **E** (**X**_enc_) with the global encoding features X_enc_ from the input image *X* is located at the left side of the model. The input of the model can be either a down scale image *X*_*G*_ or an image patch *X*_*p*_ from the original image *X*_*o*_ with high-resolution. The classification branch in the middle of the model employs the global average pooling for the extraction of the encoding features, followed by dense and soft max layers for the classification of the input image. The network is composed of three outputs: Ŷ_clas_ denotes the classification result, Ŷs_eg_ presents the tumor segmentation result, and finally Ŷ_dist_ is the multilevel high-risk tumor result. The Ŷ_clas_ determines whether the input belongs to the normal, benign, or malignant labels. Because of the complexity and challenging conditions of the X-ray images of the knee bone, this separate classification branch at the global-context level was designed. The decoding block **D** (**X**_enc_) on the right side of the model is the expanding path that maps the encoding feature into decoding feature *X*_map_ at the pixel level. For outputting tumor segmentation mask Ŷs_eg_ and multilevel distance features Ŷ_dist_, the 2D extracted features are improved by multitask learning at the pixel level between the high-risk tumor segmentation H_dis_ and the pixel-tumor segmentation H_seg._ Ŷ_seg_ with the vector size of W×H×2 is used for the classification of pixels of the input image into normal or tumor groups. Ŷ_dist_ with the vector size of W×H×5 decides the level of attention, i.e., normal, tumor, or high-risk, based on the distance to tumor in three levels of 1 to 3.

**Figure 13:**
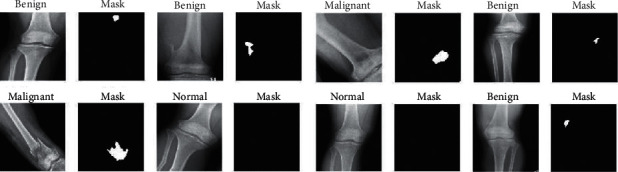
The augmented data in the global-based model underwent transform operations such as rotating, center cropping, resizing, and random flipping [78].

**Figure 14:**
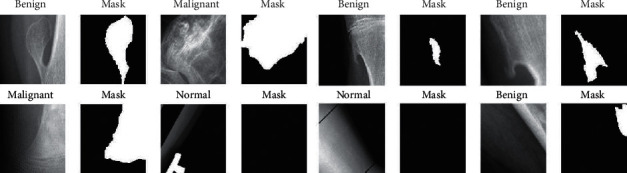
The augmented data in the patch-based model underwent transform operations such as rotating, center cropping, resizing, and random flipping [78].

**Figure 15:**
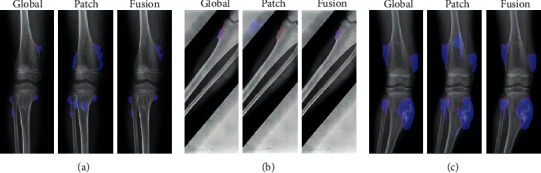
The successful ability of the global-based model to enhance the fusion results for the detection of (a) small tumors, (b) variant poses, and (c) large tumors. The red lines and the blue regions demonstrate the ground truth and tumor detection, respectively [78].

**Figure 16:**
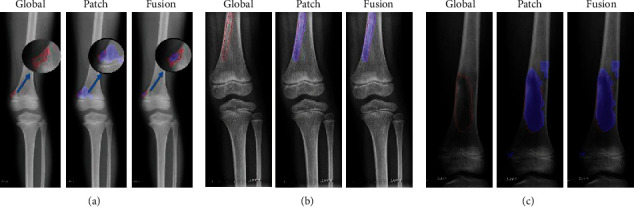
The successful ability of the patch-based model to enhance the fusion results for the detection of (a) small tumors, (b) long tumors, and (c) large tumors. The red lines and the blue regions demonstrate the ground truth and tumor detection, respectively [78].

As, in many cases, tumors are very small in comparison to the background regions, this model made an attempt to detect small tumors by learning mutual information from adjacent feature maps around tumors. The size of the high-resolution image compared to the very small size of the tumors is one of the challenges in knee bone tumor detection. Due to the memory limits, the input image is normally resized to be suitable for the global-based patch, leading to a loss of some image texture important for tumor recognition, especially for small tumors. The patch-based model that learns image texture detailed from image patches can solve this problem. The global- and patch-based models' data used to train the network are demonstrated in Figures 13 and 14. The effectiveness and the ability of each model (global-based model and patch-based model) in the detection of bone tumors are illustrated in Figures 15 and 16. Noise in small tumors, nontumor detection in variant pose, and noise in larger tumors are the failures of the patch-based model (Figure 15), whereas it assists global-based model for the detection of small, long, and large tumors (Figure 16). The Seg-UNet network proposed by Do et al. [78] demonstrated that the fusion model of the patch- and global-based models could provide mean classification accuracy of 99.05% and segmentation mean IoU of 84.84% for bone tumor segmentation and classification when both global- and patch-based models are used.

#### 2.4.2. Temporal Radiographic Texture Analysis (tRTA)

tRTA was developed as an alternative to the conventional method of diagnosis of osteolysis, in which the condition of patients is postsurgically monitored by several X-ray images [79]. As osteolysis is a biological process that evolves and appears slowly, early follow-up and diagnosis of osteolysis in the radiographic images are cumbersome and in some cases impossible tasks. Furthermore, the symptoms of trabecular texture changes caused by osteolysis are difficult to observe normally until the lesions progress. Although CT images could ameliorate the detection process of osteolysis, the cost and exposure issues make them impractical for regular follow-up. tRTA is a computerized radiographic texture analysis method used as an alternative to RTA previously used for the measurement of the patterns of trabecular bone to assist the detection of osteolytic lesions [79–81]. In the tRTA method, ROIs including the potential osteolytic lesions are selected from the image database, taken during follow-up, and visually compared with the images of the previous sessions. Then, texture features are computed from the selected ROIs to perform trend analysis with simple linear regression technique, BANN temporal analysis technique, and a LDA merging features technique. This method has the advantage of incorporating the absolute texture measures, as well as how these measures alter over time.

#### 2.4.3. Morphometry

**Figure 17:**
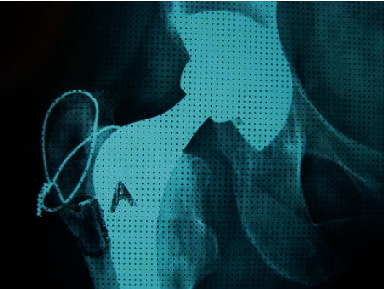
The superimposition of the morphometric grid onto the morphometry grid on a radiographic image for the measurement of osteolytic lesion areas [82].

In this method, the area of osteolytic lesions is measured based on the idea of cross intersect counting [82]. A morphometric grid is superimposed over the region of interest on the radiographic images, and then the number of test points overlapping the area of interest is counted (Figure 17).

Based on the study carried out by Smith et al. [82], the application results of morphometric method are compared with the estimations of professional orthopedists and proved to be reliable for the measurement of the area of osteolytic lesions if applied by trained orthopedic observers (nonmedical or medical health professionals).

#### 2.4.4. Software and Toolkits

Various software packages and toolkits have been developed for the purpose of medical image processing. Although they are not specifically developed for a single medical purpose, a variety of them are used by the medical profession and researchers for tumor detection and analysis using a variety of imaging modalities. The most popular and commonly used software and toolkits are mentioned as follows.

*(1) ImageJ*. This is an open-source image processing program developed in Java language inspired by NIH Image [83]. This program is currently available on all the current computer platforms and can be used with variety of plugins and macros. Although ImageJ is not specialized software, it is sometimes used by professionals and researchers for medical purposes. It performs its tasks in eight steps: dataset resizing, component hollowing, volume rendering, model slicing, image slicing, image cropping, standardization of the image size, and analysis. Nevertheless, ImageJ suffers from a variety of limitations such as a lake of 3D data analysis and requiring heavy interuser variability.

**Figure 18:**
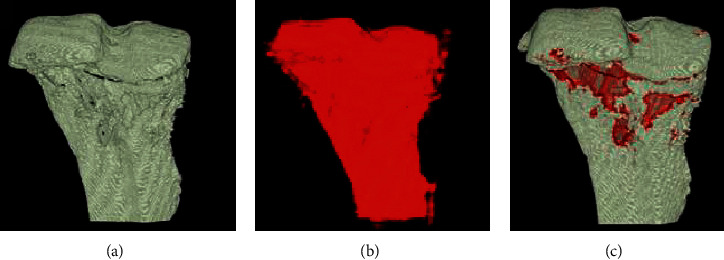
The method of calculation of osteolytic lesions in Osteolytica. (a) Original volume of the sample. (b) Reconstruction of the surface of the sample and filling lesions, diffusing the volumetric surface outwards. (c) The volume of the osteolytic lesions measured through the subtraction of the original surface from the reconstructed one. The expanded volume will diffuse inwards through a variable over the surface [84].

*(2) Osteolytica*. It is image processing software specifically designed for the measurement of lytic bone lesions [84]. It employs novel graphic card acceleration, and it is capable of 3D rendering to make a rapid analysis and reconstruction of osteolysis. It is designed to be faster, more user-friendly, and less biased compared to manual osteolytic lesion measuring methods or ImageJ 2D analysis. The principal goal of Osteolytica is to measure the dimension and areas affected by osteolysis in a 3D bone analysis. It is composed of four operating processes: dataset resizing, dataset loading, selecting the maximum lesion size, and finally lesion analysis. Osteolytica employs the process of reconstructing the surface of a volume sample and then subtracting the reconstructed volume from the original surface through volumetric diffusion method (Figure 18) [85].

**Figure 19:**
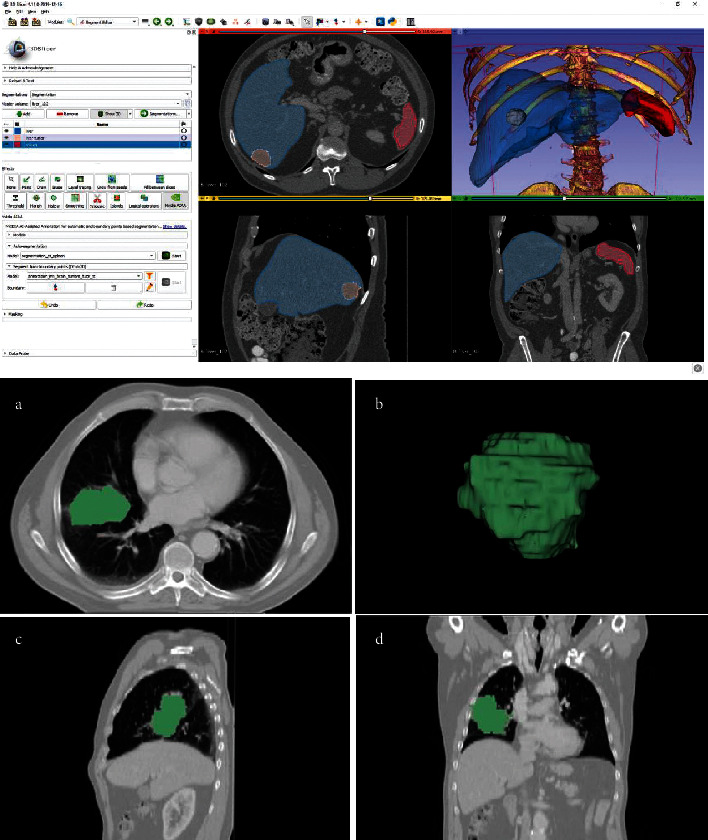
The top figure illustrates the workbench of the 3D Slicer with a variety of toolkits and tools that can be used for different purposes. The bottom figure demonstrates a typical tumor segmentation capability of the 3D Slicer [86].

*(3) 3D Slicer*. 3D Slicer is an open-source, free, and multiplatform software package that can be widely used for various medical applications such as virtual reality, real-time 3D ultrasound reconstruction, adaptive radiation therapy, tracked ultrasound for needle guidance, robot assistance intervention, surgical navigation, and image segmentation (Figure 19) [85, 87]. Furthermore, this software provides multiorgan analysis from head to toe and supports various imaging modalities including ultrasound, computed tomography, magnetic resonance imaging, microscopy, and nuclear medicine imaging. It also provides real-time analysis, which is highly useful during surgical navigation. Nevertheless, 3D Slicer is not approved for clinical use, and the distribution is intended for research use although there is no restriction on its employment.

**Figure 20:**
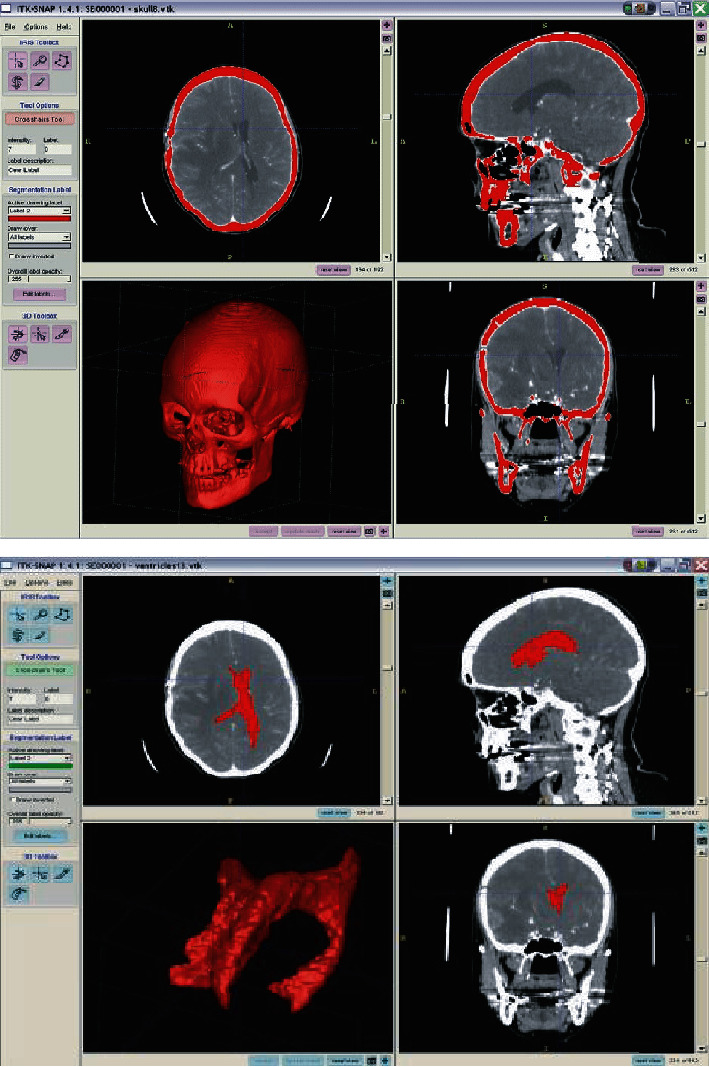
The workbench of ITK software and its segmentation ability (in this typical case, brain bone and tumor segmentation) [88].

*(4) ITK*. The Insight Toolkit (ITK) is an open-source, cross-platform library developed by the he US National Library of Medicine of the National Institutes of Health to provide developers with an extensive suite of software tools for the segmentation and registration of medical images (Figure 20) [89, 90]. ITK with its extreme programming methodologies and spatially oriented architecture allows processing medical images in two, three, or more dimensions.

**Figure 21:**
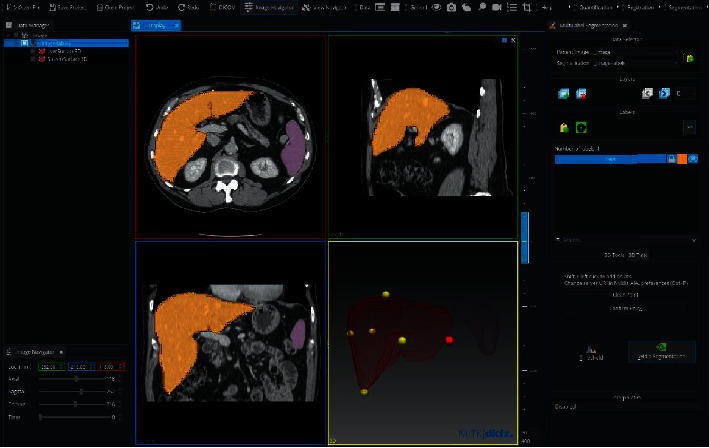
Workbench of MITK for medical image processing purposes [91].

*(5) MITK*. The Medical Imaging Interaction Toolkit (MITK) is free, open-source software for the development of interactive medical image processing software [91, 92]. MITK is a class library based on ITK that provides leading edge segmentation and registration techniques and forms the basis of algorithms. It executes the visualization commands with the Visualization Toolkit (VTK) [93]. MITK workbench has a highly customizable and extensible end-user application providing all steps of a clinical workflow such as data retrieval, image analysis, image-guided therapy, diffusion imaging treatment planning, tool tracking, diagnosis, intervention support, and treatment control (Figure 21).

### 2.5. Overview of the Image Processing Diagnosis Methods

Various techniques and methods that can be automatic, semiautomatic, or manual can be employed for the detection and segmentation of bone tumors, to be more specific osteolytic lesions. Depending on the objective of the research, they can provide different accuracies. In the previous section, the state-of-the-art automatic and semiautomatic computer-based methods, as well as manual methods with the use of software and toolkits, were mentioned for this purpose. However, the methods and techniques are not limited to this. An overview of the mentioned methods with their key points, in order to provide a well comparison for selection and application, is presented in Table 1.

### 2.6. Bone Tumor Datasets

Datasets play a prominent role in the performance of neural networks. Comprehensive datasets can well train the networks during the training phase. Subsequently, they can have a great impact on the effectiveness and accuracy of the proposed computer-based methods for the detection and segmentation of bone tumors. The larger and the more comprehensive the datasets are, the more valuable the provided data will be. It is important to note that not all the datasets are publicly available for use. Some datasets are private and require formal requests and legal permission for use. Furthermore, the images could be acquired through different imaging modalities such as MRI, CT, PET, SPECT, or conventional X-rays and with different anatomical positions that should be implemented for the evaluation of the networks based on the objective's similarities.  Table 2 presents a brief overview of the private and public datasets that can provide highly valuable data regarding bone tumors that can be used for training and evaluation of the proposed deep learning networks for the detection of osteolytic lesions.

### 2.7. Treatments for Osteolysis

Osteolytic lesions can be treated though two ways: surgical and nonsurgical treatment.

#### 2.7.1. Surgical Treatments

Operative treatments are recommended to address prior or ongoing osteolytic lesions and to correct failing articulation. Surgical treatments of osteolytic regions of THA, TJA, and shoulder arthroplasties follow a similar philosophy [100]. Nevertheless, the methods are varying depending on the structure, anatomical location, function, design, and materials employed.

If the prosthetic components are aligned, well-fixed, functional, and proper modular replacement parts can be obtained, the operative surgical treatment is then focused on the revision of the bearing surfaces and potential grafting of bone.

The osteolytic lesions caused by metal corrosion and byproducts from metal-on-metal implants are debrided, and modular metal-on-metal bearings are replaced with other ones, normally with ceramic on polyethylene [101].

#### 2.7.2. Nonsurgical Treatments

Nonoperative treatment of osteolysis is a possible option for patients who are either not able to tolerate reoperation immediately or under low loads of prosthetic byproducts and susceptible to osteolysis. The main goal of nonoperative treatment is merely to postpone the need for reoperation and also to keep the size of bone defects limited. The common nonsurgical treatment methods are mentioned as follows.

*(1) Bisphosphonates*. Bisphosphonates are the drugs that should be consumed orally or parenterally to treat metabolic disease, osteolysis associated with metastatic and osteoporosis. This medication is a synthetic analogue of pyrophosphate and is advised for osteolysis treatment in a physician-directed or off-label manner [102]. The action mechanism of bisphosphonates is mainly on the osteoclast that undergoes apoptosis leading to the inhibition of bone resorption [103, 104].

*(2) Cell Therapy*. Delivery of cells as a local therapeutic platform can indirectly or directly affect osteolysis. This can heal the bone and provide paracrine and autocrine factors [105]. Autologous bone grafting is one type of local cell therapy. Crosstalk between MSCs and macrophages is also an ongoing process in all the inflammatory bone disorders and bone healing process [106, 107].

## 3. Conclusion

Osteolysis is a progressive and biological reaction to particulate wear debris. It is the most common indication for revision surgeries after total joint arthroplasty in long-term reviews. The biological mechanisms leading to osteolytic lesions are only now beginning to be understood. Although many research studies have been carried out to characterize the complex cellular interactions that result in the bone loss, it is still not apparent why some patients undergo early osteolysis and in others this phenomenon is postponed for many years or even never occurs. It is more probable that the observed differences between the conditions of patients indicate the differential sensitivity or differences in ability to mount a wear response and generate wear particles. To expand our knowledge about the process of osteolysis, the basic science studies must be translated into clinical studies and eventually clinical practice. Although in general there is no specific classification for osteolysis, it is categorized based on the measurement of volume of the affected lesions. Studies on the genetic profile may help to explain the variability in the rate of development and the extent of osteolysis in different patients.

To detect this asymptomatic process, regular radiographic follow-up is necessary. Imaging modalities like MRI and CT scans are utilized where the extent of bone loss is uncertain. Nevertheless, it is important to note that each imaging modality has its pros and cons. In addition, various computer-based image processing methods have been developed as clinically assisted tools to facilitate this process. Among them, artificial neural networks have proved to be highly efficient for tumor detection and segmentation. The research results of the popular deep learning networks used for tumor detection such as CNN, U-Net, and Seg-UNet proved that these deep learning algorithms could assist early detection and analysis of osteolytic lesions. Further developments and studies in this field are highly crucial for the development of a solid and specialized network for the detection and segmentation of osteolysis.

There are numerous nonsurgical therapeutic interventions for the treatment of the osteolytic lesion, but they still require clinical approval to be verified. Some of them have already passed limited clinical evaluations. Nevertheless, it must be determined that progressive osteolysis resulting from wear debris is not only a biological process, but also related to modular interfaces, material issues, or failure of the bearing surface. In this respect, currently, there is no evidence to prove that nonsurgical treatment methods can clinically treat osteolysis except for delaying the process. Although it is hoped that changes in the design of implants can positively affect the reduction of wear particles and osteolysis development, only time will prove this. Fortunately, there are some strong research results supporting this issue. Nevertheless, long-term clinical follow-up is desperately required to make any new benchmarks.

## Figures and Tables

**Table 1:** A brief overview of the computer-based methods mentioned in this study for the purpose of analysis of medical images with osteolytic lesions.

Method name	Technique	Purpose of the application	Mechanism of the application
AnoGAN	Adversarial learning (generative adversarial neural network (GAN))	This unsupervised learning method is suitable when the dataset is limited	This method performs anomaly detection by generating a large number of nonlesion images by GAN to detect images with lesions.
SG-CNN	Convolutional neural network (CNN)	This method can automatically produce ROI areas independently through a superlabel-guided CNN	This method can improve classification accuracy by generating fine-grained labels and superlabels of the region of interest in medical images whose lesions of interest are not well apparent.
U-Net	U-Net structure deep neural network	This method is suitable for the segmentation of lesions when they have abnormal shape and low contrast and are susceptible to transition during classification	The U-Net structure performs semantic segmentation of the osteolytic lesions on the input image by concatenating the convolutional layers in the encoder path with the deconvolutional layers in the decoder path.
Seg-UNet	Multilevel Seg-UNet	This method is suitable for segmentation of lesions of interest on the input image when the lesion has abnormal shape and low contrast and the size of the lesion is very small compared to the input image size	The Seg-UNet exploits U-Net structure as well as the global- and patch-based approach in order to improve the classification accuracy.
tRTA	Mathematical computation	This method is a manual image processing method for the segmentation of the lesions of interest from dataset that requires trained medical practitioners	The tRTA is a computerized radiographic texture analysis method for the evaluation of ROI through linear regression, BANN temporal analysis technique, and a LDA merging features technique.
Morphometry	Manual computation	The method employs the cross-intersect counting approach	In this method, with the use of a morphometric grid that is superimposed onto the region of interest on the radiographic images, computation is performed.
ImageJ	Manual image processing	General-purpose image processing software	ImageJ can take the advantage of different plugins and macros for various image processing goals.
Osteolytica	Manual image processing	Specifically designed for the measurement of lytic bone lesions	This image processing software is designed for 3D analysis of lesions and requires trained staff.
3D Slicer	Medical image processing software	Manual image processing	3D Slicer is medical software designed only for research purposes that can perform various image analyses using variety of packages on different anatomical positions.
ITK	Open-source medical library	Manual image processing	This medical library is suitable for developers for medical image processing purposes.
MITK	Open-source medical library	Manual image processing	This class medical library is based on the ITK library and provides segmentation and registration techniques. It also has a highly customizable workbench.

**Table 2:** The names and properties of the potential datasets that can be used for training deep learning neural networks for bone tumor detection and segmentation. Each dataset is also labeled as either public or private.

Name of the dataset	Description	Type of the data	Number of images
CNUH [78] (private)	Provided by the Chonnam National University Hospital (CNUH)	CT datasets focusing on benign and malignant tumors in two regions of knee bone of distal femur and proximal tibia	Benign tumor: 1061, malignant tumor: 134, normal: 381
Shenzhen No. 2 People's Hospital [71] (private)	Derived from patients diagnosed with bone tumors in the years 2014–2017	CT images stored in DICOM	6422 images
Hokkaido University [67] (private)	Provided by the Hokkaido University in Japan	CT images of metastatic and nonmetastatic tumor images	8790 images
Sichuan University [94] (private)	Provided by the Institutional Ethics Committee of West China Hospital in Sichuan University	Bone scintigraphy (BS) images using SPECT/CT taken from patients diagnosed with bone metastasis and having undergone whole-body BS	13477 images
Public datasets [95] (public)	CT images corresponding to different body parts	CT images manually segmented	270 images
In-house dataset [96] (private)	Containing bone images of different body parts: head, chest, abdomen, neck	CT images	16218 images
Bone tumor [97] (private)	Containing benign and malignant bone tumors	Plain radiography	2899 images
CNUH [98] (private)	Provided by the Chonnam National University Hospital initially for bone segmentation on a deep learning approach containing both malignant and benign tumors	Plain radiography	963 total images, benign tumor: 329, malignant tumor: 134
DIAGNOSTIKO IATRIKI A.E. [63] (private)	Collected from prostate cancer patients with suspected bone metastatic disease, who underwent whole-body scintigraphy	Scintigraphy images (SPECT)	908 total images, 778 bone scan, 328 bone metastasis, 271 benign scan, 179 normal
Chittagong University [99] (private)	Consisting of 60 MRI images of patients diagnosed with bone cancer and their grand truth images	MRI images	60 total images, benign tumor: 30, malignant tumor: 30

## Data Availability

The authors did not use any specific dataset.
